# Intensive online contact, sleep difficulties, and overweight among Swedish adolescents: findings from the 2017/18 and 2021/22 HBSC surveys

**DOI:** 10.1186/s12889-026-27434-w

**Published:** 2026-04-21

**Authors:** Mohammad A Alhazmi, Petra Löfstedt, Maria Corell, Lauren Lissner, Monica Hunsberger

**Affiliations:** 1 School of Public Health and Community Medicine, Sahlgrenska Academy, Institute of Medicine, Gothenburg University, Box 469, 405 30, Gothenburg, Sweden; 2 Department of Medical Emergency Services, Faculty of Health Sciences, Al- Qunfudah, Umm Al-Qura University, Makkah, Saudi Arabia

**Keywords:** Health behaviour in school-aged children overweight, Family affluence scale, Vigorous physical activity and fear of missing out

## Abstract

**Background:**

Digital media use has increased rapidly among adolescents, raising concerns about its impact on health. Prior evidence suggests that intensive online contact may disrupt sleep and contribute to unhealthy behaviors that increase the risk of being overweight. This study examined associations between intensive online contact, sleep difficulties, and overweight among Swedish adolescents.

**Methods:**

We analyzed data from the Swedish Health Behaviour in School-aged Children (HBSC) surveys conducted in 2017/18 and 2021/22. The sample included 8,861 students in grades 5, 7, and 9. Intensive online contact was defined as interacting “almost all the time throughout the day” with at least one peer or social group. Outcomes included self-reported sleep difficulties, and overweight defined according to the World Health Organization’s BMI-for-age criteria. Logistic regression models estimated crude and adjusted odds ratios (OR), controlling for sex, grade, family affluence scale, unhealthy food consumption, and vigorous physical activity.

**Results:**

Intensive online contact was associated with higher odds of sleep difficulties in both crude (OR = 1.30; 95% CI: 1.16–1.45) and adjusted models (OR = 1.28; 95% CI: 1.14–1.44). Sleep difficulties were also more common in 2021/22 compared with 2017/18. No association was observed between intensive online contact and overweight after adjusting for sex, grade, Family Affluence scale, unhealthy food consumption and vigorous physical activity. (aOR = 0.96; 95% CI: 0.83–1.10). Girls reported more sleep difficulties but had lower odds of being overweight than boys.

**Conclusion:**

In this nationally representative sample of Swedish adolescents, intensive online contact was consistently associated with sleep difficulties, but not with overweight. Given evidence from previous studies linking sleep difficulties to overweight, these findings raise the possibility of longer-term implications that cannot be evaluated in cross-sectional analyses. The results align with existing recommendations to limit time spent on digital media use.

## Background

Digital media has become a defining feature of contemporary society, transforming how individuals’ access, create, and share information. Unlike traditional mass media such as print journalism, radio, or broadcast television, which rely on fixed physical infrastructures, digital platforms allow for immediacy, interactivity, and global connectivity [1, 2]. Today, approximately 67.9% of the world’s population, about 5.56 billion people, engage with digital media daily [3]. The widespread availability of smartphones, computers, and tablets has contributed to the rapid expansion of digital communication, while social media platforms have become central arenas for social interaction, entertainment, and learning among young people [4]. As accessibility has increased, screen-based media exposure has risen sharply, with adolescents aged 9–15 now spending nearly three hours per day on screens and social media which more than double the levels observed in 2010 [5].

In this study, we distinguish between related but conceptually different forms of media use. “Digital media use” is used as a broad term referring to engagement with digital devices, encompassing both passive and interactive activities. “Screen time” refers specifically to the duration of exposure to screens, regardless of activity type. In contrast, “online contact” refers to digitally mediated social interaction with peers, representing a more interactive form of media use and the primary exposure of interest in the present study. These different forms of media engagement may have distinct behavioral and health implications, with evidence suggesting that the type and context of media use may be more important than overall screen time [6].

Similar to much of the world, nearly all Swedish adolescents aged 13–16 (95%) report having a social media account, and about 84% use digital media daily [7]. Evidence suggests that screen time among Swedish children and adolescents increased between 2018 and 2021, as documented by the Generation Pep study among youth aged 4–17, although more recent data indicate a small decline in daily social media use among primary school children in 2024 [8, 9].

These trends have prompted health authorities to recommend limiting screen time; however, guidelines differ by age group. For example, the World Health Organization provides specific recommendations for younger children, whereas for adolescents the emphasis is on reducing excessive recreational screen use and promoting balanced daily behaviors. Some national guidelines additionally recommend limiting screen time to no more than two hours per day [10, 11]. A growing body of evidence links digital media use with sleep difficulties adolescents. Excessive engagement, particularly interactive online communication with peers via social media, has been associated with delayed bedtimes, shorter sleep duration, and trouble initiating or maintaining sleep [12–14]. The mechanisms underlying these associations are both physiological and psychosocial. Blue light emitted from screens suppresses melatonin, disrupts circadian rhythms, and delays the onset of sleep [15]. At the same time, peer interactions, notifications, alerts, and the fear of missing out (FOMO) encourage adolescents to check devices late at night, increasing sleep interruptions and reducing overall sleep quality [16].

Digital media exposure has also been implicated in the growing prevalence of overweight and obesity among adolescents. Globally, more than 390 million children and adolescents aged 5–19 are now overweight, including 160 million with obesity, an increase from 8% in 1990 to 20% in 2022 [17]. In Sweden, data from the self-reported Health Behaviour in School-aged Children (HBSC) study show that overweight and obesity among adolescents have more than doubled since 1989/90, with overweight increasing from approximately 7% to 15% and obesity rising from less than 1% to nearly 4% by 2017/18 [17]. Several pathways may link digital media use to excess weight gain. Screen-based activities typically promote sedentary behavior and may displace physical activity. Additionally, eating in front of screens can interfere with hunger and satiety cues, increasing caloric intake [18].Digital media also exposes adolescents to food advertising, which encourages the consumption of energy-dense, nutrient-poor foods [19].

Importantly, sleep difficulties as an outcome of excessive media use may also contribute to being overweight, providing a potential indirect pathway linking the exposure to weight outcomes [20]. Short sleep duration is associated with changes in dietary behaviors, including more frequent eating occasions, higher intake of calorie-dense foods, and increased snacking opportunities simply because adolescents remain awake for longer periods [21] .Insufficient sleep has also been linked to skipping breakfast, higher consumption of sweets and fast foods, and greater odds of overweight and obesity [22]. Longitudinal evidence from the European IDEFICS/I. Family cohort further shows that both increased screen time and shorter sleep duration independently predict incident overweight and obesity among children [20]. While previous research has primarily focused on overall screen time and general digital media use, studies have also linked increased digital engagement with adverse mental health outcomes among adolescents [23]. However, less is known about the specific role of interactive online social contact with peers. This distinction is important, as socially engaging forms of digital communication may have different implications for sleep patterns and related health behaviors than passive media consumption. Therefore, the present study aims to examine the association between intensive online contact, sleep difficulties, and being overweight among adolescents.

## Theoretical framework

Although several conceptual models address digital media use and adolescent well-being, this study draws primarily on the Displacement Theory. The Displacement Theory suggests that time spent on one activity may reduce the time available for other, potentially more beneficial behaviors. In the context of adolescent digital engagement, intensive online contact may displace time required for restorative sleep routines or physical activity, both essential for healthy development [24]. However, displacement represents only one possible mechanism, and other processes such as psychological arousal and social stimulation may also contribute to the observed associations.

This theoretical perspective is particularly relevant for sleep outcomes, as online communication frequently occurs during evening hours when adolescents are most vulnerable to disruptions in circadian rhythms and sleep onset. Engaging in social communication late at night may delay bedtime, fragment sleep, and reduce total sleep duration. The Goldilocks Hypothesis [25], proposes that moderate levels of media use may be beneficial, whereas both low and excessive use may be less optimal, suggesting potential non-linear associations. While the Goldilocks Hypothesis provides a general framework for understanding variation in outcomes across levels of media use, the present study focuses specifically on differences between levels of intensive online contact.

The Displacement Theory may also help to explain associations with weight outcomes, although the pathway is more indirect. For example, if online communication displaces physical activity or contributes to sleep disruption, this may influence energy balance and weight status. However, these pathways remain theoretical and are not directly tested in the present study. Theoretical distinctions between interactive and passive screen use suggest that not all forms of digital engagement operate through the same pathways, highlighting the importance of studying specific types of digital behavior [24].

Building on this theoretical framework, the present study hypothesizes that adolescents with higher levels of intensive online contact are expected to report higher levels of sleep difficulties, and that higher levels of online contact will also be associated with increased odds of being overweight.

Therefore, the present study examines the prevalence of intensive online contact and associations between two health outcomes, sleep difficulties and overweight, among Swedish adolescents participating in the 2017/18 and 2021/22 HBSC surveys.

## Methods

### Study design and participants

This study based upon data from the Swedish Health Behaviour in School-aged Children (HBSC) survey, a school-based and nationally representative study conducted every four years in collaboration with the World Health Organization (WHO). The Swedish HBSC is coordinated by the Public Health Agency of Sweden and Statistics Sweden. The HBSC survey uses a two-stage sampling procedure. First, schools across Sweden are randomly selected to represent the national population of school-aged children. Second, one class within each selected school is randomly chosen, and all students in that class are invited to participate. The survey targets students who are approximately 11, 13, and 15 years old, corresponding to grades 5, 7, and 9. Students complete the questionnaire anonymously during regular school hours. This design provides a broad and representative picture of the health and well-being of Swedish adolescents while keeping the data collection process manageable for schools.

The current study draws on the two most recent data collection periods. The first of these, conducted in 2017/18, involved 450 randomly selected schools across Sweden, of which 213 participated (47%). The second, conducted in 2021/22, followed the same procedure, with 247 of 450 schools participating. Statistics Sweden conducted analyses comparing participating and non-participating schools in the 2021/22 survey wave, including characteristics such as school type (public vs. private), school size, and parental educational background. These analyses did not indicate substantial differences between participating and non-participating schools [26]. A total of 8,861 (4,325 boys [50.1%] and 4,305 girls [49.9%]) students agreed to participate in the two survey periods combined. According to Statistics Sweden, it is not possible to determine the exact number of students invited. Many schools complete a “class report” indicating how many students are enrolled in the selected class and how many were absent on the day of data collection. However, not all schools complete this report. Therefore, Statistics Sweden estimated the number of invited students and calculated response rates at the level of students. Further details are provided in the technical report from Statistics Sweden [26]. Data from the 2017/18 and 2021/22 survey waves were combined to increase statistical power. Because the 2021/22 survey was conducted during/after the COVID-19 pandemic, survey year was included as a covariate in the adjusted analyses to account for potential period-related differences. At the student level, response rates were high across both survey waves. In 2017/18, the overall response rate was approximately 89%, while in 2021/22 it was approximately 80%, based on national HBSC data. These response rates indicate good representativeness of the participating samples, although some degree of non-response bias cannot be excluded. For the first part of the present analysis, we included the 8,062 adolescents who provided information on their online contact and sleep difficulties. When examining overweight, the sample reduced to *n* = 6,651 due to missing data on height or weight or implausible values (*n* = 93 reported bmi > 45). We did not perform formal comparisons between excluded and included participants in terms of demographic or socioeconomic characteristics, which should be considered when interpreting the findings.

### Measurements

### Exposure

#### Intense online contact

Intensive social media use (SMU) was measured using four questions adapted from the EU Kids Online Survey [27]. Participants were asked: How often do you have online contact with the following four groups? (1) close friends or friends. (2) friends from another circle of friends. (3) Friends I meet online. (4) people other than friends (e.g., parents, brothers/sisters, classmates, teachers). Response options ranged from 1 “do not know or not applicable” to 6 “almost all the time throughout the day”. Those who reported using social media almost all the time with any of the four groups were classified as intensive users. In contrast, all others were categorized as non-intense users according to a previous publication [27].

#### Outcomes

 Body Mass Index (BMI) was determined based on the 2007 growth reference standards set by the WHO for children and adolescents aged 5 to 19 years. Standardized z-scores were used to calculate BMI based on self-reported height and weight. This approach accounts for age- and sex-specific variation in BMI and does not use adult BMI cut-offs. Overweight was coded as equivalent to BMI values at + 1 standard deviation (SD), and obesity was coded as equivalent to BMI values at or above + 2 SD [28]. For the analysis, overweight and obesity were combined into a single category ( ≥ + 1 SD) and compared with normal weight. Individuals with implausible values, BMI under 11 or over 45 were excluded according to the protocol [29].

 Sleep difficulty was assessed by a single item on the HBSC symptom checklist: “In the last 6 months, how often have you had difficulties in getting to sleep?”. This item was answered on a five-point scale: about every day, more than once a week, about every week, about every month, and rarely or never. Answers were dichotomised as having sleep difficulties “about every day or more than once a week” [1] or not (0) [30].

## Confounders/covariates

### Family affluence scale (FAS III)

The socioeconomic status of the respondents and their families was evaluated using a six-question assessment of a family’s absolute wealth level. 

The questions include:

1. Does your family own a car or another motorized vehicle? (No = 0; Yes, one = 1; Yes, two or more = 2).
2. Do you have your own bedroom? (No = 0; Yes = 1).
3.  How many computers (including laptops and tablets, but not game consoles and smartphones) do your family own? (None = 0; One = 1; Two = 2; More than two = 3).
4. How many bathrooms (rooms with a bath/shower or both) are in your home? (None = 0; One = 1; Two = 2; More than two = 3).
5. Does your family have a dishwasher? (No = 0; Yes = 1).
6. How many times did you and your family travel out of Sweden for holiday/vacation last year? (Never = 0; Once = 1; Twice = 2; More than twice = 3).

The responses are summed and then categorized as follows: 0 − 7 (low FAS), 8 − 11 (medium FAS), and 12 − 13 (high FAS) These categories follow common HBSC practice and are based on the relative distribution of FAS scores within the sample rather than fixed absolute cut-offs [31, 32].

### Unhealthy food consumption

Participants’ eating behaviors were assessed using a food-frequency questionnaire, which asked how often they typically consumed sweets and soft drinks over a week. Response options ranged from “never” to “more than once per day”. Each response was converted into a numerical value to count weekly intake: “never” was coded as 0, “less than once a week” as 0.5, “once a week” as 1, “2–4 days per week” as 3, “5–6 days per week” as 5.5, “once a day” as 7, and “more than once a day” as 8. Scores for sweets and soft drinks were then summed up to produce a total weekly consumption score, with a possible range from 0 to 16. This approach is consistent with previous HBSC studies, where similar items are combined to capture overall consumption of energy-dense, nutrient-poor foods and beverages. Combining these items provides an indicator of general unhealthy dietary patterns, which are relevant for studying associations with health outcomes in adolescent populations. To support further analysis, this total score was dichotomized at the median value of the sample: participants with scores below the median were classified as having low consumption, and those at or above the median were classified as having high consumption [33].

### Vigorous physical activity

The measure of vigorous leisure-time physical activity was derived from the question, “how often do you usually exercise in your free time so much that you get out of breath or sweat?”. Response options included: “Never,” “Less than once a week,” “Once a week,” “2–3 times a week,” “4–6 times a week,” and “Every day.” For analysis, the variable was dichotomized by grouping participants reporting vigorous physical activity 4–6 times per week or daily versus those reporting less frequent activity. This categorization was chosen to approximate current World Health Organization recommendations that children and adolescents should engage in at least 60 min of moderate-to-vigorous physical activity daily, although the HBSC measure reflects frequency rather than duration and therefore provides an approximation rather than a direct measure of adherence to the guidelines [11, 34].

### Statistical analysis

Descriptive statistics were used to summarize sample characteristics by survey year. Logistic regression models were applied to estimate crude odds ratios (ORs) and adjusted odds ratios (aORs) with 95% confidence intervals (CIs) for the associations between intensive online contact and sleep difficulties, and between intensive online contact and overweight. Adjusted models controlled for survey year, sex, grade, family affluence, and unhealthy food consumption in all analyses. Vigorous physical activity was additionally included in models examining being overweight. Survey year was included as a categorical covariate in all models to account for period effects. To examine whether the association between intensive online contact and sleep difficulties differed by weight status, stratified logistic regression analyses were conducted by overweight status (normal weight vs. overweight). Analyses were conducted using complete-case data, including only participants with non-missing values on all variables included in the models. To account for clustering of observations within classes, additional analyses were conducted using generalized estimating equations with an exchangeable correlation structure. All analyses were conducted using SPSS.

## Results

Table 1 summarizes sample characteristics presented by survey year, 2017/2018 and 2021/2022. In total, 8,861 students are included in the analysis. The prevalence of intensive online contact decreased from 1655 (41.9%) in 2017/2018 to 1450 (35.3%) in 2021/2022. These differences are descriptive and were not formally tested for statistical significance. Reports of sleep difficulties increased from 1245 (30.4%) in 2017/2018 to 1655 (37.1%) in 2021/2022. Weight status remained stable, with approximately 80% of participants classified as normal weight in both survey years. Dietary patterns showed an increase in unhealthy food consumption, increasing from 53.4% to 63.0%. In contrast, levels of vigorous leisure-time physical activity ranged from approximately 36% to 39% of adolescents reporting activity four or more times per week in both survey periods.

**Table 1:** Sociodemographic and health characteristics of the study sample

Survey year	2017/2018	2021/2022
Participants *n* = 8,861	**N (%)**	**N (%)**
	4294(48.5)	4567 (51.5)
Intensity of online contact *n* = 8,062		
Non-intensive contact	2297 (58.1)	2660 (64.7)
Intensive contact	1655 (41.9)	1450 (35.3)
Weight status (BMI) *n* = 6,651		
Normal weight	2754 (80.3)	2576 (80.0)
Overweight	676 (19.7)	645 (20.0)
Sleep difficulty *n* = 8,556		
No difficulty	2855 (69.6)	2801 (62.9)
Difficulty sleeping	1245 (30.4)	1655 (37.1)
Sex *n* = 8,630		
Boy	2101(49.8)	2224 (50.2)
Girl	2114 (50.2)	2191 (49.8)
Grades *n* = 8,861		
Grade 5	1181 (27.5)	1559 (34.1)
Grade 7	1452 (33.8)	1575 (34.5)
Grade 9	1661 (38.7)	1433 (31.4)
Family Affluence Scale (FAS) *n* = 8,505		
Low	657 (16.0)	1000 (22.8)
Medium	2895 (70.4)	3050 (69.4)
High	559(13.6)	344 (7.8)
Unhealthy food consumption *n* = 8,525		
Low consumption	1911 (46.6)	1631 (36.9)
High consumption	2194 (53.4)	2789 (63.1)
Vigorous physical activity in leisure time (VPA) *n* = 8,548		
Up to 2–3 times a week (No)	2639 (63.9)	2682 (60.7)
4–6 times a week or more (Yes)	1493 (36.1)	1734 (39.3)

Table 2 presents the logistic regression estimates for sleep difficulties. Intensive online contact was associated with higher odds of sleep difficulties than those with non-intensive contact in both crude (OR = 1.29; 95% CI: 1.17–1.42) and adjusted analyses (aOR = 1.28; 95% CI: 1.16–1.42). Girls reported higher odds of sleep difficulties compared with boys (aOR = 1.56; 95% CI: 1.41–1.73). Grade level showed an inverse pattern: both grade 7 (aOR = 0.82; 95% CI: 0.73–0.93) and grade 9 students (aOR = 0.86; 95% CI: 0.76–0.98) had lower odds of sleep difficulties compared with grade 5 students. Family affluence was not significantly associated with sleep difficulties after adjustment for potential confounding variables.

**Table 2:** Crude and adjusted associations between intense online contact and sleep difficulty among Swedish adolescents (2017/18 and 2021/22)

Variables	Crude OR (95% CI) Sleep difficulty	*Adjusted OR (95% CI) Sleep difficulty
Intensity of online contact		
Non-intensive user (ref.)	1.00	1.00
Intensive user	1.29 (1.17–1.42)	1.28 (1.16–1.42)
Survey year		
2017/2018 (ref.)	1.00	1.00
2021/2022	1.35 (1.23–1.48)	1.27 (1.15–1.41)
Sex		
Boy (ref.)	1.00	1.00
Girl	1.53 (1.40–1.68)	1.56 (1.41–1.73)
Grades		
Grade 5 (ref.)	1.00	1.00
Grade 7	0.90 (0.80-1.00)	0.82 (0.73–0.93)
Grade 9	0.90 (0.80–1.09)	0.86 (0.76–0.98)
Family Affluence Scale (FAS)		
Low (ref.)	1.00	1.00
Medium	0.87 (0.78–0.99)	0.89 (0.78–1.01)
High	0.83 (0.70-1.00)	0.90 (0.75–1.10)
Unhealthy food consumption		
Low consumption (ref.)	1.00	1.00
High consumption	1.29 (1.18–1.42)	1.30 (1.17–1.44)

* Mutually adjusted for all variables shown

Reference category for outcome: no sleep difficulties

Table 3 presents ORs and aORs with 95% CIs for the association between overweight status and selected variables. In the crude model, intensive online contact was associated with significantly lower odds of being overweight compared to non-intensive contact (OR = 0.86; 95% CI: 0.75–0.97), although this association was reduced and became non-significant in the adjusted model (aOR = 0.94; 95% CI: 0.81–1.08). The survey year was not significantly associated with overweight in either the crude (OR = 1.02; 95% CI: 0.90–1.15) or the adjusted analysis (aOR = 0.95; 95% CI: 0.8–1.08). Girls exhibited lower odds of overweight than boys in both crude (OR = 0.60; 95% CI: 0.53–0.67) and adjusted models (aOR = 0.59; 95% CI: 0.49–0.64). Grade level also demonstrated a significant crude association, with grade nine students having lower odds of being overweight compared to grade five students (OR = 0.79; 95% CI: 0.68–0.92), though the association was weaker in the adjusted model (aOR = 0.83; 95% CI: 0.70–1.00). Family affluence was inversely associated with being overweight; among medium and high affluence families showed lower odds compared to low affluence families in both crude (OR = 0.68; 95% CI: 0.58–0.79 and OR = 0.49; 95% CI: 0.39–0.63, respectively) and adjusted models (aOR = 0.64; 95% CI: 0.54–0.76 and aOR = 0.47; 95% CI: 0.36–0.61, respectively). Both unhealthy food consumption and vigorous physical activity were not significantly associated with being overweight. Sleep difficulty was also tested in the models; however, its inclusion did not change the results, and it was therefore not included in (Table 3).

**Table 3:** Crude and adjusted associations with overweight among Swedish adolescents (2017/18 and 2021/22)

Variables	Crude OR (95% CI) Overweight	*Adjusted OR (95% CI) Overweight
Intensity of online contact		
Non-intensive user (ref.)	1.00	1.00
Intensive user	0.86 (0.75–0.97)	0.94 (0.81–1.08)
Survey year		
2017/2018 (ref.)	1.00	1.00
2021/2022	1.02 (0.90–1.15)	0.94 (0.82–1.08)
Sex		
Boy (ref.)	1.00	1.00
Girl	0.60 (0.53–0.67)	0.56 (0.49–0.64)
Grades		
Year 5 (ref.)	1.00	1.00
Year 7	0.89 (0.76–1.02)	0.88 (0.75–1.04)
Year 9	0.79 (0.68–0.92)	0.83 (0.70-1.00)
Family Affluence Scale (FAS)		
Low (ref.)	1.00	1.00
Medium	0.68 (0.58–0.79)	0.64 (0.54–0.76)
High	0.49 (0.39–0.63)	0.47 (0.36–0.61)
Unhealthy food consumption		
Low consumption (ref.)	1.00	1.00
High consumption	0.93 (0.82–1.06)	0. 89 (0.78–1.02)
Vigorous physical activity in leisure time (VPA)		
Up to 2–3 times a week (No) (ref.)	1.00	1.00
4–6 times a week or more (Yes)	0.99 (0.87–1.12)	1.08 (0.95–1.24)

* Mutually adjusted for all variables shown

Reference category for outcome: not overweight (normal weight)

Table 4 presents stratified analyses by weight status, showing that intensive online contact was associated with increased odds of sleep difficulties in both normal-weight and overweight adolescents, with similar effect estimates.

**Table 4:** Stratified adjusted associations between intensity of online contact and sleep difficulties by weight status among Swedish adolescents

Intensity of online contact	Normal weight * (Adjusted OR ,95% CI)	Overweight * (Adjusted OR ,95% CI)
Non-intensive user (ref.)	1.00	1.00
Intensive user	1.27 (1.12–1.45)	1.34 (1.04–1.74)

*Mutually adjusted for sex, grade, cohort, FAS and Unhealthy food behaviours

## Discussion

This study examined associations between intensive online contact and two health outcomes, sleep difficulties and overweight, among Swedish adolescents, using nationally representative data from 2017/18 to 2021/22. The study confirms that Intensive online contact was associated with higher odds of reporting sleep difficulties, a finding consistent with earlier research showing that heavy digital use contributes to delayed bedtimes, shorter sleep duration, and difficulties initiating sleep [12, 30].

Differences between the 2017/18 and 2021/22 survey waves should be interpreted with caution, as the latter was conducted during/after the COVID-19 pandemic. Survey year was included as a covariate to account for period-related differences; however, broader contextual effects may not be fully captured. Alternative approaches, such as multilevel modeling, could be considered in future research. Several mechanisms may explain this relationship, including melatonin destruction from blue light exposure, displacement of bedtime routines, and the stimulating nature of online social interactions [15]. However, these mechanisms were not directly measured in the present study and should therefore be interpreted as potential explanations rather than empirically tested pathways. While the findings are consistent with the idea that higher levels of online contact may be associated with poorer sleep outcomes, they do not directly test the non-linear associations proposed by the Goldilocks Hypothesis and should therefore be interpreted with caution. They are also consistent with Displacement Theory, which proposes that intensive digital contact displaces time needed for restorative nighttime routines. Similarly, Displacement Theory may also explain why too much digital engagement may displace time for physical activity. However, the lack of an association between intensive online contact and being overweight in this study suggests that the pathways linking digital engagement to weight outcomes may be more complex. Although Displacement Theory provides a possible explanation, these mechanisms were not directly tested in the present study, and physical activity was not significantly associated with being overweight in the adjusted models. Therefore, the role of displacement in explaining weight-related outcomes remains theoretical [24, 25].In contrast, the study did not find an association between intensive online contact and being overweight. This may seem unexpected given well-established evidence linking screen time to adolescent obesity. However, the specific measure used, involving intensity of online communication, captures a form of screen engagement that differs from more passive behaviors strongly associated with weight gain, such as television viewing or prolonged sedentary screen time combined with snacking. Prior studies emphasize that the type and context of screen use are important, with passive modalities showing stronger connections to obesogenic behaviors [19, 35]. Sleep-related mechanisms may also help explain these findings. Inadequate sleep has been shown to disrupt appetite regulation, increase snacking frequency, and influence dietary choices and overall energy expenditure [36, 37]. Short or poor-quality sleep has likewise been associated with reduced physical activity and higher daytime fatigue, which can contribute to a higher energy intake [38]. However, the degree of sleep disruption associated with intensive online communication and its potential influence on dietary behaviors or physical activity were not directly measured in this study [39]. Importantly, stratified analyses indicated that the association between intensive online contact and sleep difficulties was observed in both normal-weight and overweight adolescents, suggesting that weight status does not modify this relationship [20].

Sociodemographic differences were also observed. Girls were less likely to be overweight but more likely to report sleep difficulties, consistent with previous studies recording gender patterns in adolescent health [40, 41]. Youth from more affluent families were less likely to be overweight, showing that children from higher-affluence families are less exposed to obesogenic environments, partly because they have greater access to healthier foods, structured leisure activities, and opportunities for physical activity [42]. By contrast, affluence played a less significant role in predicting sleep problems, suggesting that late-night online use reduces across socioeconomic groups [43]. These findings have meaningful implications for public health recommendations. Our finding support recommendations for sleep, interventions should focus on timing and bedtime routines, such as encouraging digital time limit, muting notifications, and reducing evening exposure to bright screens [44].For overweight, strategies may be better directed to the food environment and eating habits during screen use, rather than limiting social communication itself [18]. Identifying that digital media has outcome-specific effects may help design more effective and acceptable public health strategies.

A strength of this study is that it draws on large-scale data from a nationally representative sample of Swedish students in grades 5, 7, and 9. Another strength is that the instruments used in the survey are validated and widely applied within the HBSC protocol, supporting the reliability of the measures. However, several limitations should be acknowledged. Data was self-reported, which may introduce recall and reporting biases. BMI was based on self-reported height and weight, and sleep was assessed with a single item, which does not capture all aspects of sleep quality. However, this measure has been widely used in HBSC studies and adolescent health research. The cross-sectional design prevents conclusions about causality, and our measure of online contact did not differentiate between time of day, content, or purpose of use, which may be important moderators. In addition, several variables were dichotomized for analytical purposes, which may have led to some loss of information and reduced sensitivity to detect dose–response relationships, although this approach facilitates interpretation and comparability with previous HBSC research. Another limitation concerns the construction of the dietary variable. Sweets and soft drinks were combined into a single measure to capture overall consumption of energy-dense, nutrient-poor foods. While this approach is consistent with previous HBSC research, these items may differ in their consumption patterns and socioeconomic associations, which may have influenced the observed relationships and potentially masked more specific associations. Future research should build on these findings by using longitudinal designs to explore temporal relationships and causal pathways. Differentiating between passive and interactive forms of screen use, as well as examining bedtime-specific behaviors, will help explain mechanisms. Investigating mediators such as physical activity and peer influence will also be significant. Such work would contribute to a more precise understanding of how digital media affects sleep disturbances and could inform the development of modified interventions.

## Conclusion

Intensive online contact was associated with higher odds of sleep difficulties but showed no association with being overweight. These findings suggest that different forms of digital engagement, particularly socially interactive use, may relate differently to health outcomes rather than having uniform effects. Recognizing this complexity is important for designing effective interventions, and efforts to improve sleep may benefit from focusing on patterns of online social interaction and healthy digital routines, while strategies to address overweight may need to prioritize food environments and eating behaviors. Future longitudinal research should continue to examine the diverse ways in which intensive online contact affects adolescent well-being, ensuring that public health approaches are targeted, evidence-based, and relevant to young people’s everyday digital lives.

## Data Availability

No datasets were generated or analysed during the current study.

## Abbreviations

aOR: Adjusted odd ratio
BMI: Body mass index
CI: Confidence interval
HBSC: Health behaviour in school-aged children
FAS: Family affluence scale
FOMO: Fear of missing out
OR: Odd ratio
OW: Overweight
SD: Standard deviation
SMU: Social media use
VPA: Vigorous physical activity
WHO: World health organisation

## References

[1] Strauss C, Harr MD, Pieper TM. Analyzing digital communication: a comprehensive literature review. Manage Rev Q. 2025;75:3119–57.

[2] Han L. The rise of digital media: transforming communication, culture, and commerce. Global Media J. 2024;22(72):427.

[3] Kirksekiz A, Yildiz M, Kiyici M, Emrem M, Koroglu S. Adults social media addiction and phubbing latent profiles and authenticity as outcome. BMC Psychol. 2025;13(1):580. 40448203 10.1186/s40359-025-02858-y PMC12124037

[4] Klinger D, Plener PL, Marboe G, Karwautz A, Kothgassner OD, Dienlin T. Exploring the relationship between media use and depressive symptoms among gender diverse youth: findings of the Mental Health Days Study. Child Adolesc Psychiatry Mental Health. 2024;18(1):104. 10.1186/s13034-024-00797-x PMC11342596 39175045

[5] European Commission Joint Research Centre. Why are children and adolescents vulnerable to social media? Luxembourg: European commission joint research centre. 2025. Available from: https://joint-research-centre.ec.europa.eu/jrc-explains/why-are-children-and-adolescents-vulnerable-social-media_en. Accessed 3 Apr 2026.

[6] Orben A, Przybylski AK. The association between adolescent well-being and digital technology use. Nat Hum Behav. 2019;3(2):173–82. 30944443 10.1038/s41562-018-0506-1

[7] Beeres DT, Andersson F, Vossen HG, Galanti MR. Social media and mental health among early adolescents in Sweden: a longitudinal study with 2-year follow-up (KUPOL study). J Adolesc Health. 2021;68(5):953–60. 32943289 10.1016/j.jadohealth.2020.07.042

[8] Delisle Nyström C, Carlander A, Cassel S, Rosell M, J-Son Höök M, Löf M. Physical activity and screen time in Swedish children and adolescents: the generation pep study 2018–2021. Acta Paediatr. 2023;112(3):460–8. 36371645 10.1111/apa.16594 PMC10098717

[9] Folkhälsomyndigheten, Sociala medier Folkhälsomyndigheten. 2024.Available from: https://www.folkhalsomyndigheten.se/publikationer-och-material/publikationsarkiv/h/how-do-you-talk-about-screens-at-home-leaflet/. Accessed 27 Mar 2026.

[10] Chaput J-P, Willumsen J, Bull F, Chou R, Ekelund U, Firth J, et al. 2020 WHO guidelines on physical activity and sedentary behaviour for children and adolescents aged 5–17 years: summary of the evidence. Int J Behav Nutr Phys Activity. 2020;17(1):141. 10.1186/s12966-020-01037-z PMC7691077 33239009

[11] World Health Organization. WHO guidelines on physical activity and sedentary behaviour. Geneva: World Health Organization; 2020. 33369898

[12] Bozzola E, Spina G, Agostiniani R, Barni S, Russo R, Scarpato E, et al. The use of social media in children and adolescents: scoping review on the potential risks. Int J Environ Res Public Health. 2022;19(16):9960. 36011593 10.3390/ijerph19169960 PMC9407706

[13] Gariepy G, Danna S, Gobiņa I, Rasmussen M, de Matos MG, Tynjälä J, et al. How are adolescents sleeping? adolescent sleep patterns and sociodemographic differences in 24 European and North American countries. J Adolesc Health. 2020;66(6):S81–8. 32446613 10.1016/j.jadohealth.2020.03.013

[14] Khan A, Reyad MAH, Edwards E, Horwood S. Associations between adolescent sleep difficulties and active versus passive screen time across 38 countries. J Affect Disord. 2023;320:298–304. 36183824 10.1016/j.jad.2022.09.137

[15] Alam M, Abbas K, Sharf Y, Khan S. Impacts of blue light exposure from electronic devices on circadian rhythm and sleep disruption in adolescent and young adult students. Chronobiology Med. 2024;6(1):10–4.

[16] Bozzola E, Spina G, Ruggiero M, Vecchio D, Caruso C, Bozzola M, et al. Media use during adolescence: the recommendations of the Italian Pediatric Society. Ital J Pediatr. 2019;45(1):149. 31775828 10.1186/s13052-019-0725-8 PMC6880642

[17] Folkhälsomyndigheten. Overweight and obesity among school children 11–15-year-old continues to increase. Solna and Östersund; 2020. pp. 20020–1. Report No.

[18] Jensen ML, Carpentier FRD, Corvalán C, Popkin BM, Evenson KR, Adair L, et al. Television viewing and using screens while eating: associations with dietary intake in children and adolescents. Appetite. 2022;168:105670. 34478756 10.1016/j.appet.2021.105670 PMC8671257

[19] Robinson TN, Banda JA, Hale L, Lu AS, Fleming-Milici F, Calvert SL, et al. Screen media exposure and obesity in children and adolescents. Pediatrics. 2017;140(Suppl 2):S97–101. 29093041 10.1542/peds.2016-1758K PMC5769928

[20] Guzman V, Lissner L, Arvidsson L, Hebestreit A, Solea A, Lauria F, et al. Associations of sleep duration and screen time with incidence of overweight in European children: the IDEFICS/I.Family cohort. Obes Facts. 2022;15(1):55–61. 34724664 10.1159/000519418 PMC8820133

[21] Deng X, He M, He D, Zhu Y, Zhang Z, Niu W. Sleep duration and obesity in children and adolescents: evidence from an updated and dose–response meta-analysis. Sleep Med. 2021;78:169–81. 33450724 10.1016/j.sleep.2020.12.027

[22] Tambalis KD, Panagiotakos DB, Psarra G, Sidossis LS. Insufficient sleep duration is associated with dietary habits, screen time, and obesity in children. J Clin Sleep Med. 2018;14(10):1689–96. 30353810 10.5664/jcsm.7374 PMC6175799

[23] Twenge JM, Joiner TE, Rogers ML, Martin GN. Increases in depressive symptoms, suicide-related outcomes, and suicide rates among US adolescents after 2010 and links to increased new media screen time. Clin Psychol Sci. 2018;6(1):3–17.

[24] Hall JA, Liu D. Social media use, social displacement, and well-being. Curr Opin Psychol. 2022;46:101339. 35395533 10.1016/j.copsyc.2022.101339

[25] Przybylski AK, Weinstein N. A large-scale test of the goldilocks hypothesis: quantifying the relations between digital-screen use and the mental well-being of adolescents. Psychol Sci. 2017;28(2):204–15. 28085574 10.1177/0956797616678438

[26] Statistics Sweden (SCB). Skolbarns hälsovanor 2021–2022: Teknisk rapport. Internal report. Stockholm: Statistics Sweden (SCB); 2022. Report No.: 898838-8/255301.

[27] Boer M, Van Den Eijnden RJ, Boniel-Nissim M, Wong S-L, Inchley JC, Badura P, et al. Adolescents’ intense and problematic social media use and their well-being in 29 countries. J Adolesc Health. 2020;66(6):S89–99. 32446614 10.1016/j.jadohealth.2020.02.014 PMC7427320

[28] Mercedes de Onis a, Adelheid W, Onyango a C, Siekmann J. Development of a WHO growth reference for school-aged children and adolescents. Bull World Health Organ. 2007;85(9):660-7. 10.2471/BLT.07.043497 PMC2636412 18026621

[29] Venäläinen J, Låftman SB, Landberg J. Weight status and psychosomatic complaints in Swedish adolescent boys and girls: does family support play a buffering role? BMC Public Health. 2024;24(1):3024. 39482648 10.1186/s12889-024-20517-6 PMC11529216

[30] Khan A, Thomas G, Karatela S, Morawska A, Werner-Seidler A. Intense and problematic social media use and sleep difficulties of adolescents in 40 countries. J Adolesc. 2024;96(5):1116–25. 38570320 10.1002/jad.12321

[31] Jonsson KR, Bailey CK, Corell M, Löfstedt P, Adjei NK. Associations between dietary behaviours and the mental and physical well-being of Swedish adolescents. Child Adolesc Psychiatry Mental Health. 2024;18(1):43. 10.1186/s13034-024-00733-z PMC10981827 38555430

[32] Boer M, Moreno-Maldonado C, Dierckens M, Lenzi M, Currie C, Residori C, et al. The implications of the COVID-19 pandemic for the construction of the family affluence scale: findings from 16 countries. Child Indic Res. 2024;17(1):395–418.

[33] Borraccino A, Lemma P, Berchialla P, Cappello N, Inchley J, Dalmasso P, et al. Unhealthy food consumption in adolescence: role of sedentary behaviours and modifiers in 11-, 13-and 15-year-old Italians. Eur J Pub Health. 2016;26(4):650–6. 27085192 10.1093/eurpub/ckw056 PMC5885947

[34] Lorente JRS, Cutillas MD, Rivas SL, de Los Santos FJR, Diaz AN, Monteagudo-Piqueras O. Factors associated with physical activity engagement among adolescents in a southeastern region of Spain. Prev Med Rep. 2025;53:103063. 40256409 10.1016/j.pmedr.2025.103063 PMC12008529

[35] Haghjoo P, Siri G, Soleimani E, Farhangi MA, Alesaeidi S. Screen time increases overweight and obesity risk among adolescents: a systematic review and dose-response meta-analysis. BMC Prim Care. 2022;23(1):161. 35761176 10.1186/s12875-022-01761-4 PMC9238177

[36] Doan N, Parker A, Rosati K, van Beers E, Ferro MA. Sleep duration and eating behaviours among adolescents: a scoping review. Health Promotion Chronic Disease Prev Canada: Res Policy Pract. 2022;42(9):384. 10.24095/hpcdp.42.9.02 PMC9559193 36165765

[37] Chaput J-P, Dutil C. Lack of sleep as a contributor to obesity in adolescents: impacts on eating and activity behaviors. Int J Behav Nutr Phys Activity. 2016;13(1):103. 10.1186/s12966-016-0428-0 PMC5037605 27669980

[38] Duraccio KM, Krietsch KN, Chardon ML, Van Dyk TR, Beebe DW. Poor sleep and adolescent obesity risk: a narrative review of potential mechanisms. Adolesc Health Med Ther. 2019;10:117–30. 31572040 10.2147/AHMT.S219594 PMC6749827

[39] Cal-Herrera A, Corbella-González A, Climent-Llinares S, Fernández-Rodríguez OI. The impact of social media on adolescents’ eating and sleeping habits: a systematic review and meta-analysis. Healthcare. 2025;13(22):2962. 41302350 10.3390/healthcare13222962 PMC12652198

[40] Widome R, Lenk KM, Laska MN, Erickson DJ, Iber C, Kilian G, et al. Sleep duration and weight-related behaviors among adolescents. Child Obes. 2019;15(7):434–42. 31290691 10.1089/chi.2018.0362 PMC6761589

[41] Eriksson M, Lingfors H, Golsäter M. Trends in prevalence of thinness, overweight and obesity among Swedish children and adolescents between 2004 and 2015. Acta Paediatr. 2018;107(10):1818–25. 29637596 10.1111/apa.14356

[42] Sares-Jäske L, Grönqvist A, Mäki P, Tolonen H, Laatikainen T. Family socioeconomic status and childhood adiposity in Europe: a scoping review. Prev Med. 2022;160:107095. 35594926 10.1016/j.ypmed.2022.107095

[43] Lewien C, Genuneit J, Meigen C, Kiess W, Poulain T. Sleep-related difficulties in healthy children and adolescents. BMC Pediatrica. 2021;21(1):82. 10.1186/s12887-021-02529-y PMC7885393 33593333

[44] Hale L, Kirschen GW, LeBourgeois MK, Gradisar M, Garrison MM, Montgomery-Downs H, et al. Youth screen media habits and sleep: sleep-friendly screen behavior recommendations for clinicians, educators, and parents. Child Adolesc Psychiatr Clin N Am. 2018;27(2):229–45. 29502749 10.1016/j.chc.2017.11.014 PMC5839336

